# The GRoNC: Guidelines for Reporting on Norm-Referenced and Criterion-Referenced Scores

**DOI:** 10.1177/10731911251371395

**Published:** 2025-09-24

**Authors:** Marieke E. Timmerman, Annelies De Bildt, Julian Urban

**Affiliations:** 1 University of Groningen, The Netherlands; 2 Accare Child and Adolescent Psychiatry, Groningen, The Netherlands; 3 GESIS Leibniz Institute for the Social Sciences, Mannheim, Germany; 4 Trier University, Germany

**Keywords:** assessment, psychological test, reporting standards, standardized scores, test interpretation

## Abstract

Psychological test manuals vary widely in their reporting of the construction and interpretation of standardized scores. Consequently, the critical evaluation of norm quality and meaning is difficult for test users and reviewers. Because a specific standard for reporting on standardized scores is lacking, we developed Guidelines for Reporting on Norm-referenced and Criterion-referenced Scores (GRoNC), following a systematic approach for creating reporting guidelines (EQUATOR). The development took place in two stages: Stage 1, developing a preliminary version of the GRoNC based on a literature review; Stage 2, a Delphi process in two rounds, involving both theoretical experts (*n =* 11) and test developers (*n =* 14). The GRoNC includes a series of questions and associated explanations. It supports test developers in developing and reporting upon their standardized scores, and reviewers in evaluating a psychological test on its standardized scores. We provide recommendations on using the GRoNC and conclude by describing our expectations and plans to increase the impact of the GRoNC on reporting practice.

Standardized psychological tests are widely used, both in individual assessment and in research. Individual assessments are carried out in clinical, developmental, and human resource practice, to facilitate individual diagnosis, monitoring, and (job and education) selection. Standardized tests lead to a standardized test score, which allows for interpreting the score of the individual. One distinguishes two types of standardized test score interpretations, namely norm-referenced and criterion-referenced interpretations (American Educational Research Association et al., 2014, pp. 95–96). In a norm-referenced interpretation, like an intelligence test, the standardized test score expresses an individual’s performance in relation to a particular reference population. Examples of norm-referenced scores are percentiles, *T*-scores, and normalized IQ-scores. In a criterion-referenced test, like a behavioral screener, the standardized test score expresses a comparison to a specified level of performance (e.g., Reynolds & Livingston, 2019, pp. 53–54). A criterion-referenced score is categorical, or ordinal, like level of impairment in the categories none, moderate, and severe.

The standards for educational and psychological testing (American Educational Research Association et al., 2014) provide criteria for the development and evaluation of tests. Yet, the standards that pertain to the construction and interpretation of standardized test scores (Ch. 5, Ch. 7 Cluster 2) are rather general. For example, Standard 5.2 states that the procedures and their rationale for deriving the standardized test scores should be described clearly, such that users can judge their quality and precision. Specific guidance on how this needs to be done is lacking. Some important aspects can be inferred from subsequent standards (e.g., Standard 5.8, which refers to defining the normative population), but this is far from complete. Various review systems exist for evaluating test quality (e.g., Diagnostik-Und Testkuratorium, 2018; EFPA, 2013; Evers et al., 2009) that are more specific than the standards for educational and psychological testing. Yet, these review systems too lack coverage of several crucial aspects. For example, recent developments in effective and efficient methodology for calculating norms in norm-referenced tests (continuous norming; e.g., Innocenti et al., 2023; Lenhard et al., 2018; Timmerman et al., 2021) are not included. Thus, there is no up-to-date, comprehensive, and specific standard for how to develop and report upon the construction of standardized test scores. As a result, psychological test manuals vary widely in what and how they report (Urban et al., 2024b). This hampers a critical evaluation of the quality and interpretation of the standardized test scores involved and, consequently, a correct interpretation of an individual’s standardized test score.

To aid test constructors and test reviewers in considering all relevant aspects of standardized test scores, this article presents Guidelines for Reporting on Norm-referenced and Criterion-referenced Scores (GRoNC). Moreover, test constructors can use the GRoNC for making informed decisions in the process of developing suitable standardized test scores for their psychological test. With the GRoNC, we aim to stimulate the consistent and complete reporting of well-underpinned choices to arrive at high-quality standardized test scores, as well as the correct interpretation of a standardized test score. Thus, we aim to promote both the development of high-quality standardized test scores and high-quality interpretation of those test scores.

The GRoNC has been developed following the systematic approach for the creation of health reporting guidelines (Moher et al., 2010), as advocated by the EQUATOR Network (n.d.). We implemented a so-called “reactive” Delphi method (McKenna, 1994), where experts responded to a previously constructed version of the GRoNC (rather than generating these themselves). The EQUATOR approach is summarized in a checklist (Moher et al., 2010). In Appendix A of the Supplementary Material on the project’s OSF page,^1^ we present this checklist in columns one and two and indicate whether and how we applied each item, including a rationale, in column three. Below, we first describe the process of developing the GRoNC. Then, we present the GRoNC itself, which includes questions and a detailed explanation of these questions. We conclude by discussing how to use the GRoNC.

## The Development of the GRoNC

We developed the GRoNC for two main reasons. First, prominent existing guidelines (American Educational Research Association et al., 2014; Diagnostik-Und Testkuratorium, 2018; EFPA, 2013; Evers et al., 2009) lack comprehensiveness regarding the estimation of standardized test scores. As a result—and second—tests vary widely in how the standardized test scores are developed, and what and how their manuals report on the standardization procedure. More specifically, there is considerable variation in the approach taken, the terminology used and the level of detail provided in test manuals. This issue was mentioned by Lenhard et al. (2019) for tests involving continuous norming, reflecting our own anecdotal experiences with test manuals, and is empirically supported by a review of German test manuals (Urban et al., 2024b). Inconsistent approaches, inconsistent terminology, and insufficient detail hinder the critical evaluation of the standardized scores and hence psychological diagnostics. A comprehensive guideline with a consistent and precise terminology could therefore help to improve the reporting on standardized test scores and, as a result, help the user to better understand and evaluate what is reported. In developing the GRoNC, we aimed for such a clear, structured tool for psychological test constructors to use while developing standardized test scores and preparing the associated sections of a test manual, including their technical justification and their interpretation. The tool would also facilitate reviewers in evaluating a psychological test with respect to their standardized scores.

The process of developing the GRoNC took place in two stages: Stage 1, aiming at developing a preliminary version of the GRoNC; Stage 2, a Delphi process in two rounds, involving theoretical experts in the first round and test developers in the second.

### Researcher Description

Timmerman has published on statistical models for psychological test norming, has been involved in the development and norming of different psychological tests, and is a member of a national committee evaluating test quality. De Bildt has investigated the psychometric properties of several instruments for autism and intellectual disabilities and has been involved in developing norms for specific target populations. Urban is involved in the development of cognitive tests, has examined the psychometric properties of international measurement instruments, and has published on continuous norming.

### Stage 1

This stage contained two steps, that is, a literature review and the actual writing of the preliminary version of the GRoNC.

Step 1, the literature review, involved a systematic literature search to underpin our claims of a lacking comprehensive guideline and varying standardization approaches and terminology and to achieve a well-founded basis for developing a comprehensive guideline. More specifically, we aimed to (1) identify whether there is a comprehensive guideline and (2) review the literature on standardized test score construction and interpretation, using the database PsycInfo in April to May 2023. The literature review aimed at identifying the prevalent theoretical perspectives on standardized test score construction and their practical use and interpretation. In Appendix B of the Supplementary Material (available on OSF [see Note 1]), details on the literature review can be found. We searched for books, peer-reviewed articles, and electronic collections. We used search terms related to (a) testing, guidelines, construction, and norming, in three syntaxes to adequately span the literature while keeping the number of results manageable and (b) handbook, psychological assessment, neuropsychology, clinical psychology, special needs education, and human resource management, in one syntax. The four syntaxes resulted in a total of 1,458 titles. Titles and abstracts were screened by an assistant of Timmerman for (1) recommendations for reporting on the construction and interpretation of standardized test scores and (2) theory on the construction and interpretation of standardized test scores, possibly including guidance. Results were discussed among Timmerman, De Bildt, and Urban.

Regarding aim (1), one study (Lenhard et al., 2019) provided recommendations for the evaluation and documentation of continuous norming procedures. This study also raised concerns about how test manuals report on continuous norming (i.e., standardization) and that insufficient reporting hampers the critical evaluation of the resulting standardized test scores. The authors provided three key recommendations for evaluating and documenting continuous norms. These three recommendations refer to (a) reporting descriptive statistics of the raw score distributions to inform the selection of the statistical model for the normative data, (b) quantifying the deviation between observed and fitted data, and (c) visually inspecting the fitted percentile curves. While these are undoubtedly important aspects to report, the study has a limited focus on reporting on the modeling of continuous norms. The study excludes other crucial elements of standardization, such as criterion-referenced test scores and the procedures for collecting the sample data. Thus, a comprehensive guideline for reporting on norm-referenced and criterion-referenced scores remained lacking.

Thirty-eight sources met criterion (2) and were fully screened by Timmerman. This revealed two additional sources meeting criterion (2) via backward searching, resulting in 40 sources for full screening. Details on the search and its results are supplied in Appendix B of the Supplementary Material (available on OSF [see Note 1]). Of these 40 sources on standardized test score construction and interpretation, nine had a psychometric point of view (e.g., Hogan & Tsushima, 2016; Hunsley & Allan, 2019; Reynolds & Livingston, 2019) and 31 had an assessment point of view (e.g., Frick & Cornell, 2003; Hale et al., 2016; Horn et al., 2013). The psychometric texts typically included descriptions of the process to construct and/or interpret standardized test scores. The assessment texts differed widely in focus, including, for example, assessment in specific populations (e.g., African American adults [Freedman & Manly, 2018]), assessment related to specific settings (e.g., mental health [Horn et al., 2013], and gender identity in assessment [Brabender, 2022]). Given our purpose to develop guidelines for reporting on standardized scores, we focused on the parts related to constructing and interpreting standardized test scores.

In the 40 sources, we noticed a clear common ground in the approach to—and the interpretation of—standardized test scores. We noticed also much variety, both in aspects considered (e.g., whether concerning norm-referenced and/or criterion-referenced scores) and in terminology. The first issue is not necessarily problematic, because the area of focus differed much between the sources. The second issue is problematic, because inconsistent terminology can be confusing for readers and may yield erroneous thinking. This hampers well-founded norming practices and standardized test scoring interpretation.

**Figure 1.**
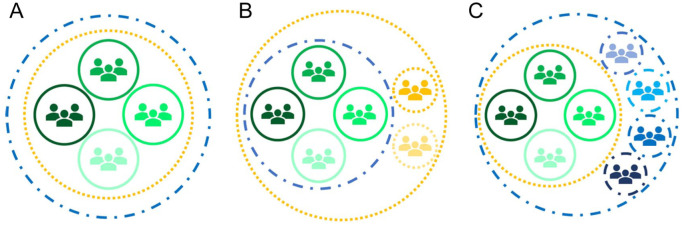
Three Typical Relationships Between the Target Population (Blue/Dashed Line), Reference Populations (Green/Full Line), and Normative Population (Yellow/Dotted Line)

We will here provide one example of confusing terminology to clarify why inconsistent terminology forms an issue. Three types of populations associated with a norm-referenced test are often mixed up. The *target population* is the specific collection of individuals to which the test may be administered. Second, the *reference population* is the population to which the standardized test scores relate, like the total population of a specific country of the same age as the individual tested. Third, the *normative population* is the complete population on which the norms are based (i.e., from which the normative sample is drawn). As depicted in Table 1 and Figure 1, the target, reference, and normative populations may not coincide in some cases. Confusing these populations, however, may result in that the test may be used for persons outside the target population (e.g., a 13-year-old person in the Panel B example). Thus, distinguishing target, normative, and reference populations using consistent terminology is important for correct test application and test score interpretation.

**Table 1.** Examples for Three Typical Relationships Between Target, Normative, and Reference Population—Accompanying Figure 1.

Panel	A	B	C
Relation between populations	• Target population equals the normative population • Each reference population is a subset of the target population and the normative population	• Target population is a subset of the normative population • Each reference population is a subset of the target population	• Normative population is a subset of target population • Each reference population is a subset of the normative population
Example	Intelligence test	Intelligence test	Neuropsychological test
Target population	Total population of a country aged 15-30 years	Total population of a country aged 15-30 years	Population of individuals who are suspected to suffer from a certain condition
Normative population	Target population (i.e., Total population of a country aged 15-30 years)	Total population of a country aged 13-32 years (i.e., target population plus 2 years beyond the test’s age boundaries)^a^	Total population of a country excluding individuals suffering from the certain condition
Reference populations	Persons of equal age within the target population	Persons of equal age within the target population	Persons of equal age, educational level, and gender within the normative population

a Extending the normative population beyond the test’s age boundaries is useful to achieve sufficient norm precision near the age boundaries.

Step 2 was the actual writing of the preliminary version of the GRoNC. Timmerman extracted themes and associated terminology based on the literature review and grouped them according to five themes: (1) the nature of the test’s target population and of the raw scores, and the required type(s) of standardized test score interpretation; (2) the nature of the test’s reference populations; (3) the study to gather sample data; (4) calculating the norms; (5) reporting. De Bildt commented upon the list, and Timmerman adapted it and wrote a first version of questions and explanations. De Bildt and Urban provided feedback, and the version was adapted over five rounds until all authors agreed to the preliminary version of the GRoNC (GRoNC-V0; available on OSF [see Note 1]), consisting of questions and explanations, including literature references and clarifications of confusing terminology.

### Stage 2

This stage was a reactive Delphi process in two rounds. In Appendix C of the Supplementary Material (available on OSF [see Note 1]), details on this stage can be found. In the first round, we invited 14 theoretical experts via three organizations involved in the assessment of tests, and by directly contacting two authors of relevant, high-quality chapters from our literature review. We sent out the GRoNC-V0 (available on OSF [see Note 1]) and asked the experts four questions: (1) Are the topics relevant to norming comprehensibly covered? If not, what topic(s) do you miss?; (2) Are the topics and questions well-explained and clear? If not, what is insufficiently explained, or unclear?; (3) Do you have any other suggestions for the GRoNC?; (4) How would you describe your expertise? In total 11 experts provided their responses, by answering the questions explicitly (eight experts) and/or by providing suggestions in the text (six experts). From these responses, Timmerman extracted 10 points to discuss with De Bildt and Urban. The points included questions like whether one can or should formulate one’s preference, as for continuous norming, whether criterion-referenced scores can be sensibly developed based on norm-referenced scores, what to advise test constructors in case the described ideal steps are not possible in practice, and suggestions to improve the presentation by adding figures and tables. Based on the discussion of the points and taking into account all suggestions made by the experts, Timmerman rewrote GRoNC-V0, with contributions from Urban. This version was adapted over two rounds until all authors agreed to the next version GRoNC-V1.

In the second round, we invited 20 experienced test developers from our own network—of whom four were employed at a test publisher, 14 at a university, and two self-employed. We strived for a reasonable spread in test area (i.e., including experts in intelligence, personality, and clinical tests) and in geographical area. We sent out the GRoNC-V1 (available on OSF [see Note 1]), and asked the experts four questions: (1) Are the topics and questions well-explained and clear? If not, what is insufficiently explained, or unclear?; (2) Do you think you would be able to write the parts related to norming for a test manual using the GRoNC? If not, could you described what is hampering?; (3) Do you have any other suggestions for the GRoNC?; and (4) How would you describe your expertise?

In total 14 experts provided their responses, by answering the questions explicitly (8 experts), or providing a few remarks (3 experts), and/or providing extensive suggestions in the text (6 experts). Those eight who responded to the questions, responded positively to questions 1 and 2 (i.e., partly, mostly, or just yes), with specific suggestions to clarify and improve. From these responses, Timmerman extracted 11 points to discuss with De Bildt and Urban. The points included questions like whether preregistration for a norming study would be helpful to improve the quality, about our explanations (e.g., of the statistical models for norming, and of the target, norm and reference populations), and suggestions to improve the presentation by visualizations. Based on their specific feedback with respect to the framing of the statistical models for norming, the team decided to ask for an additional discussion meeting with two experts, which took place with Timmerman. Based on the discussions, Timmerman rewrote GRoNC-V1, with contributions from Urban, thereby also taking into account all textual suggestions made by the experts. This version was adapted over three rounds until all authors agreed to the final GRoNC, which we present here.

## The GRoNC: Questions and Explanation

The GRoNC consists of a series of questions and associated explanations, which support test developers to evaluate and decide on their steps in the development of norm-referenced and/or criterion-referenced scores, and in reporting on their data and decisions; it also facilitates reviewers in evaluating a psychological test with respect to their standardized scores.

The questions are organized along steps: Step 1 for all tests, Steps 2–4 for each type of interpretation (i.e., A. norm-referenced and B. criterion-referenced), and Step 5 for reporting, again for all tests. We first present the questions, followed by a step-wise detailed explanation of each question, including references and examples, and discuss variants of terminology that we found in the literature.

### Questions of the GRoNC

**Table:** 

Step	A. For norm-referenced scores	B. For criterion-referenced scores
1	*Define the test’s target population, the raw scores, and the required type(s) of standardized test score interpretation* 1. Is/are the target population(s) of the test properly defined, in line with the test’s aim and including appropriate inclusion and exclusion criteria? 2. Is it described how exactly the raw test scores are obtained? 3. Is/are the selected type(s) of standardized test score interpretation(s) (i.e., norm-referenced and/or criterion-referenced) in line with their associated test aim(s)?	
2	*Define the test’s reference population(s) and the normative population* A4. Is/are the reference population(s) properly defined, in line with the test’s aim(s), including appropriate inclusion and exclusion criteria, and, if applicable, explicit description of the norm predictor(s) that define the reference populations? a. If there are multiple reference populations, is it clearly explained for which test purpose which reference population needs to be used? A5. Is the normative population (or: normative populations, in case of multiple test aims with each their own set of reference populations) properly defined?	*Select the approach to set the cut scores and the required decisions* B4. Is/are the selected approach(es) to set the cut scores (i.e., empirically based or based on norm-referenced scores) in line with their associated test aim(s)? B5. Are the required terms for the selected approach to set the cut scores properly specified, in view of the aim of the test? a. For empirically derived cut scores, these terms are the criterion, possible populations for which different cut scores may apply, and the procedure to determine the cut score(s) based on the test and criterion scores. b. For cut scores based on norm-referenced scores, these terms are the test’s reference population(s), the test’s normative population, and the percentile(s) to set the cut score(s).
3	*Design and carry out the study to gather the sample data* A6. Is the sampling design appropriate, such that it sufficiently guarantees well-estimated norms? a. Is the resulting sample sufficiently representative of the normative population, thereby considering 1. the sampling approach (i.e., random sampling, quota sampling, or stratification) and 2. the degree to which the sample distribution differs from the normative population (in terms of predictor and background variables)? b. Is the type of the norm-referenced score (e.g., z-scores, normalized z-scores, percentiles) properly described and motivated, including the procedure to calculate it? c. Is the resulting sample size and sampling scheme sufficient to achieve minimally required precision in norms, for all reference population(s), given the (expected) required (regression) model(s)? d. Does the study have a positive ethical review? If yes, where? e. Is the data collected under the required standardized testing conditions? f. Is the data collected sufficiently recent?	*Design and carry out the study to gather the sample data* B6. Is the sampling design appropriate, i.e., such that it sufficiently guarantees well-estimated cut scores? a. For empirically derived cut scores: i. Is/are the population(s) considered to determine the cut scores appropriate and sufficient to assess the quality of the settled cut scores in view of the aim of the test’s use? ii. Is the resulting sample representative of the relevant population(s)? iii. Is the resulting sample size sufficient to achieve minimally required precision in setting the cut scores? iv. Does the study have a positive ethical review? If yes, where? v. Is the data collected under the required standardized testing conditions? vi. Is the data collected sufficiently recent? b. For cut scores based on norm-referenced scores: See Questions A6a-e under Step 3A.
4	*Based on the normative sample data, settle the norms* A7. a. Does the (regression) model used for the norming provide a reasonable approximation to the reference population’s distributions? That is, is the modeling strategy well-described, with respect to . . . i. the candidate distribution(s) of the test score (if applicable), ii. the (smooth) relationship of the norm predictor(s) to the (parameters of the) test score distribution (if applicable), iii. the adjustment methods applied to correct for non-representativeness (if applicable), iv. the assumptions associated with the model involved, and to what extent they seem to be met, v. the model selection strategy (e.g., based on a fit criterion that penalizes model complexity, to prevent overfitting), and vi. the assessment of (local) model fit to the observed data (e.g., via visualizations, such as a worm-plot and centile plots)? b. If a continuous norm predictor, like age, is involved and the norms are provided in tables: Are the age intervals defining the age groups used to present the norm tables justified (i.e., as large as possible, provided that the transition in norms between adjacent age groups as well as the within-group bias is sufficiently small [Urban et al., 2024a])? A8. Are confidence intervals (e.g., 95%) provided for the norm-referenced scores? A9. Is the interpretation of the norm-referenced score and its associated confidence interval properly clarified?	*Based on the empirical data, determine the cut score(s), validate the cut scores, and describe the interpretation* B7. Are the cut score(s) properly calculated? a. For cut scores based on norm-referenced scores: See Questions 7, 8 and 9 under Step 4A. b. For empirically derived cut scores: Has the procedure to determine the cut score been followed? B8. Is the accuracy of the settled cut scores assessed via validation sample(s) from the relevant population(s)? B9. Is the interpretation of the categorization resulting from the cut score properly clarified?
5	*Reporting* 10. Is the construction of the standardized test scores clear? a. Is the construction properly described and sufficiently motivated? b. Are the analyses scripts and, if possible, (anonymized) data made available? 11. Are instructions provided for clear and useful score reports for the stakeholders and settings for which the test is intended to be used? 12. Has feedback in terms of completeness and practical usefulness from a selection of potential test users on the draft manual been incorporated?	

### Detailed Explanation of the GRoNC-Questions

#### Step 1

Define the Test’s Target Population, the Raw Scores, and the Required Type(s) of Standardized Test Score Interpretation

The target population (cf. Question 1) is the population of individuals to which the test may be administered in practice. The target population needs to be defined as explicitly and precisely as possible, using inclusion criteria (e.g., the total population of a certain country within a certain age range) and exclusion criteria (e.g., intellectual disability). The required type(s) of standardized test scores must match the intended use(s) of the test.

A standardized test score of a tested individual is derived from the raw test score (cf. Question 2) obtained by this tested individual. It needs to be described how exactly raw test scores are obtained. If weighted sum scores are used, include the weights and describe how these were obtained. If latent variable estimates are used, describe the model and specify the estimation algorithm.

There are two types of standardized test score interpretations (cf. Question 3). In a norm-referenced interpretation, the standardized test score (i.e., the norm-referenced score) expresses an individual’s raw test score relative to the raw test scores of the reference population at hand, for example, the total population of a specific country of the same age. Knowing the norm-referenced score thus supports the description of an individual’s functioning relative to the reference population, for example, pertaining to the intellectual functioning or adaptive functioning (e.g., Reynolds & Livingston, 2019, p. 47). In contrast, in a criterion-referenced interpretation, the standardized test score expresses a comparison to some judgmental standard, without making any direct reference to the raw test scores of others. This supports qualifying an individual, often in terms of a grouping, like passing or failing a certification exam, being labeled as depressed or not depressed, or being categorized on various proficiency levels on an achievement test (e.g., Hogan & Tsushima, 2016, p. 51).

Note that a single test may have both types of standardized test score interpretations, supporting a norm-referenced interpretation (e.g., normalized *T*-score) and a criterion-referenced interpretation (e.g., level of impairment [none, moderate, severe]; Hogan & Tsushima, 2016, p. 51). Furthermore, this theoretical distinction is not always sharp in practice (American Educational Research Association et al., 2014, p. 96). A norm-referenced score may be used as a basis for a criterion-referenced interpretation, for example, when using a neuropsychological test for diagnosis, as is explained in more detail in Step 2B.

##### Variations in Terminology Used in Step 1

The terms population, sample, and group are used imprecisely. A population refers to the complete set of all people we want our results to generalize to. A population could be, for example, all inhabitants of a certain country within the age range 15–30 years with a good command of the country’s language. A sample is a subset of individuals from a certain population of whom we have data. Thus, for example, a normative sample pertains to the set of individuals of whom test data are collected for the norming. A group is a collection of individuals. Because group can thus refer to both a population and a sample, the term group is confusing in this context. In the sources reviewed, the term group was often used, without clear explanation on what the authors referred to (e.g., Cagigas & Manly, 2014; Hays, 2013; Hogan & Tsushima, 2016).

### For Norm-Referenced Scores

#### Step 2A. Define the Test’s Reference Population(s) and the Normative Population

A norm-referenced score expresses an individual’s raw test score relative to the raw test scores of the reference population at hand, for example, the total population of a certain country of the same age as the tested individual (cf. Question A4). It is often needed to define exclusion criteria for the reference population. These can pertain to a minimal level of ability to carry out the test (e.g., intellectually, in vision, in hearing) and to specific conditions. For example, for a questionnaire measuring level of depression, it may be more informative to exclude individuals with a diagnosis of depression from the reference population, because then the norm-referenced score expresses the individual’s position relative to non-depressed individuals. The exclusion criteria should be defined such that the resulting reference population is in line with the aim of the test.

Norms need to be developed for each reference population specified for a test. This means that per reference population, a norm-referenced score is settled for each possible raw test score. The reference population should be specified such that it is useful in view of the aim of the test. A test may have multiple aims, suitable for different contexts, and therefore a single test may have multiple reference populations (American Educational Research Association et al., 2014, p. 97; Hunsley & Allan, 2019, p. 14).

We provide three examples of cases with multiple reference populations. First, raw scores on intelligence tests change across the lifespan and typically increase in performance across childhood, peak in early adulthood, and subsequently decline. In a selection context, the aim is to identify individuals that score in a certain range (e.g., high, medium, or low) relative to others, irrespective of their age. In an educational advisory setting, the aim is to assess the performance level of the child relative to their peers of the same age. Therefore, it makes sense to have available as the reference populations both the total population within a certain age range and the total population of the same age, so the user can choose the reference population that matches their aim of the testing.

Second, raw scores on the Strengths and Difficulties Questionnaire (SDQ; Goodman, 1997) are known to relate to age and gender. The typical use of the SDQ is for screening among children and adolescents on psychosocial issues. Because the relationship with age presumably reflects natural child development, it makes sense to have age-dependent norms. For gender, the choice appears to be more difficult to make. An argument in favor of gender-age-dependent norms is that they account for typical differences between boys and girls in experiences of psychosocial problems, notably that boys experience higher rates of conduct disorders and girls experience higher rates of emotional problems (e.g., Zahn-Waxler et al., 2008). If we would accept these as given, a high conduct score would be more alarming for a girl than for a boy. However, an argument against having gender-dependent norms is that the use of gender-specific norms would imply that one removes these natural occurring differences in the prevalence of psychosocial problems (Frick & Cornell, 2003, p. 269). A high conduct score would then be interpreted as evenly alarming, irrespective of whether this score stems from a boy or a girl. The key is which, age-dependent and/or gender-age-dependent norms, would provide the most meaningful reference in (clinical) practice, thus for the purpose of the test at hand.

Third, in a neuropsychological context, the aim is often to assess the loss in functioning due to a (presumed) brain impairment. In such a context, ideally, the reference would be the individual’s premorbid level. In the absence of this information, the “healthy” population (i.e., total population minus the individuals with a neuropsychological brain disorder) with demographic characteristics as similar as possible to the individual is the alternative reference population. The idea is that based on the known relationships between the raw test scores and the demographic variables among the healthy population, one could correct for these demographic characteristics and thus achieve good diagnostic accuracy (Freedman & Manly, 2018, p. 109).

To establish which demographic variable(s) to use, three issues are important. First, for a demographic variable to be of use to identify the reference population (and thus to correct for), it needs to be related to the raw test score distribution. Otherwise, the norms would be invariant, irrespective of the level of the demographic variable involved. Variables that have been shown to relate to certain (neuropsychological) tests are age, gender, educational level/school type, socioeconomic status, urbanization, language, race, and ethnicity (Reynolds & Mason, 2009, pp. 205–206).

Second, because each combination of categories of demographic variables identifies one reference population, one should take care of their number in relation to the sample design (see Step 3A), to ensure that the norms can be settled with sufficient precision. If certain (combinations of) demographic variables are strongly associated, it can be better to leave out certain variables. Third, to include a demographic variable, their scoring needs to be unambiguous. This means that the scoring categories need to be clearly defined.

We do advocate against the use of variables such as race and ethnicity to define the reference population, because it is neither needed nor effective to do so, while there is a serious societal risk of stigmatization. It is not needed, because the explanatory power of these variables over variables such as socioeconomic status and educational level is typically none to small (e.g., Organisation for Economic Co-operation and Development (OECD), 2023). It is ineffective, because their categorizations are based on social norms, may be fluid over time and place, and still involve a large heterogeneity among individuals within the same category (Freedman & Manly, 2018, pp. 109–110). The latter heterogeneity is observed, for example, in recent German results of the Programme for the International Assessment of Adult Competencies, where considerable differences in performances were found among those not born in Germany (Rammstedt et al., 2024).

The normative population (cf. Question A5) is the complete population on which the norms are based (i.e., from which the normative sample is drawn). The normative population entails the joint of the reference populations (for a particular test aim) and possibly a bit beyond (see Panel C of Figure 1, for example, to include in the normative population a sample of individuals beyond the age range of the test).

##### Variations in Terminology Used in Step 2A

Here, we will present variations of terminology used in Step 2A, which we found in our literature study. The term reference population (or group) is used in various sources (e.g., American Educational Research Association et al., 2014; Butcher et al., 2013; Paltzer, 2018). Alternatives are comparison group (American Educational Research Association et al., 2014), normative comparison group (Frick & Cornell, 2003), statistically relevant normative group (Groth-Marnat & Wright, 2016a), and appropriate normative group (Weiler et al., 2019).

The term normative population is used in different sources (e.g., Brabender, 2022; Freedman & Manly, 2018; Hale et al., 2016). Alternatives are standardization population (Cagigas & Manly, 2014; Groth-Marnat & Wright, 2016a; Suzuki et al., 2005) and norm population (Streiner, 2021).

The term norms is used in various sources (e.g., Brabender, 2022; Freedman & Manly, 2018; Hale et al., 2016). Alternatives are normative data (e.g., Horn et al., 2013; Mitrushina et al., 2005; Paltzer, 2018; Pedraza, 2018), normative standards (Freedman & Manly, 2018), and normative information (Goldstein et al., 2019).

The term norm-referenced score is used by Groth-Marnat and Wright (2016b). Alternatives are norm-referenced scale score (American Educational Research Association et al., 2014), norm-referenced standard score (Groth-Marnat & Wright, 2016a), norm-based score (Freeman & Chen, 2019), norm score (Gary et al., 2023; Lenhard et al., 2018), scale score (Kolen & Hendrickson, 2013), scaled score (Hambleton & Zenisky, 2013), and standardized measure (Conant, 2014).

Many sources did not distinguish the target population, reference population, and normative population, by not mentioning the target population at all (e.g., Freedman & Manly, 2018; Hays, 2013; Reynolds & Mason, 2009), or even stating that the target population and normative population ideally would equal each other (Wasserman & Bracken, 2012, p. 52). As this depends on the case at hand, it is important to distinguish these three kinds of populations.

#### Step 3A. Design and Carry Out the Study to Gather the Normative Sample Data

The norms are estimated based on the data collected in the normative sample. To achieve well-estimated norms (i.e., to model the empirical relations in the population with regard to the measured variable well enough) for all reference populations, the normative sample must be well-composed. This pertains to both the representativeness of the sample and to the sample design. A well-composed sample is achieved by applying a good sampling design, based on sample survey design methodology (Kolen & Hendrickson, 2013, p. 208; e.g., Valliant et al., 2018). We will first discuss sampling approaches to achieve a representative sample, and then the sample design needed to achieve sufficient precision.

##### Sampling Approach (cf Question A6a)

Ideally, the sampling approach involves some form of random sampling. By doing so, the statistical properties of the resulting samples are known (e.g., the probability of obtaining a representative sample increases with increasing sample size). Then, default statistical analyses can be applied. Examples are simple random sampling, stratified random sampling, and hierarchical random sampling. In normative practice, it is typically impossible to achieve a random sample, because of various hampering factors. That is, the members of the full population may be unknown (as with individuals suffering from a certain condition), data protection rules may preclude access to the members of the total population (as with the registered population of a country), and refusal to participate of sampled individuals may lead to observed data associated with a non-random sample.

When random sampling cannot be used, test constructors often resort to quota sampling, which is a non-probability sampling technique (Wasserman & Bracken, 2012, p. 54). In quota sampling, one defines quota based on a target distribution of auxiliary variables. Auxiliary variables are individual characteristics that relate to the test score. For a set of auxiliary variables to be selected for a quota sampling scheme, it is needed that their population statistics (i.e., their joint distribution) for the reference populations are available (Valliant et al., 2018, p. 1), for example, via a national statistics agency. Auxiliary variables often used in a norming context are age, educational level, immigration background, gender, geographic region, and socioeconomic status. Auxiliary variables include the so-called norm predictor(s), which are those variable(s) that identify the reference population. Thus, if the reference population consists of individuals of the same age and gender, then age and gender are the norm predictors. Any other auxiliary variables are denoted as background variables. When applying quota sampling, one needs to include at least one background variable, apart from possible norm predictors. The possible effect of the norm predictors is accounted for when computing the norms, and thus, these are not effective in achieving a representative sample. Taking into account the minimally required sample size (as discussed in the next section), the quotas are defined for each possible combination of (categorized) scores on the auxiliary variables, such that the resulting sample distribution (approximately) matches the joint distribution of the normative population with respect to the auxiliary variables. Participants are sought based on their scores on the auxiliary variables until all quotas are reached.

To provide an example, suppose that the reference populations are defined by age and that educational level is used as a background variable. Ideally, a quota sampling scheme would be determined based on the joint distribution of age and educational level in the normative population. The quotas are then defined for consecutive age intervals per educational level, such that the sample distribution of educational level per age interval matches their population distribution. If this joint distribution is unknown, one can determine the quota based on the marginal distributions of age and educational level only, and hope that the resulting empirical joint distribution mimics the population joint distribution to a reasonable extent. Note that representativeness in terms of age is not needed to achieve a good test score distribution estimate per reference population (i.e., age). Yet, the precision of the estimates may be lower in regions of the norm predictor with relatively smaller sample sizes (De Vries et al., 2025).

##### Sample Design

The sample design for a normative study involves setting the sample size and the sampling scheme (i.e., how the sample needs to be spread across the values of the norm predictors and background variables), such that one achieves sufficient norm precision. The sample size and the sampling scheme need to be based on how the norms are settled. As this relates to the type of norm-referenced scores, and how the norms are calculated, we first discuss these issues, with a summary of the key points in Table 2.

**Table 2.** Key Points of Methods to Settle the Norms in Case of Norm-Referenced Scores.

Topic/type of norm-referenced scores	Distribution shape-invariant transformation	Distribution shape-enforcing scale transformation
From raw score to norm-referenced scores	Via a linear transformation of the raw scores	Via a monotone, typically nonlinear, transformation
Examples of norm-referenced scores	*Z*-scores (μ = 0; σ = 1), IQ-scores (μ = 100; σ = 15), *T*-scores (μ = 50; σ = 10), Wechsler scaled scores (μ = 10; σ = 3)	Percentiles, deciles, stanines, normalized *Z*-scores, normalized IQ-scores, normalized *T*-scores, normalized Wechsler scaled scores
Measurement level	Minimally ordinal; interval level if the raw scores are at interval level	Ordinal, unless interval level can be motivated (e.g., if the norm-referenced scores comply with a specific IRT model)
Method to calculate the norms in case of a single, or very few reference populations	Estimate the mean and standard deviation per reference population separately, or model the mean and standard deviation as a function of the norm predictors using regression	Rank-based normalization method (e.g., Blom, Rankit, Tukey) or a continuous norming method
Method to calculate the norms in case of multiple reference populations	Model the mean and standard deviation as a function of the norm predictors using regression	Continuous norming method (e.g., cNORM, GAMLSS-based norming)

##### Type of Norm-Referenced Scores (cfQuestion A6b)

There are different ways to transform raw scores to norm-referenced scores. Herewith, we distinguish two main types, namely distribution shape-invariant and distribution shape-enforcing scale transformations (Mellenbergh, 2011, pp. 351–357). A distribution shape-invariant transformation is a linear transformation of the raw scores to scores with a predefined mean and standard deviation, thus leaving the distributional shape itself unchanged. Examples are a linear transformation of the raw scores resulting in *Z*-scores (μ = 0; σ = 1), IQ-scores (μ = 100; σ = 15), *T*-scores (μ = 50; σ = 10), or Wechsler scaled scores (μ = 10; σ = 3). As the shape of the distribution is unchanged, the linearly transformed scores are only normally distributed if the raw scores were already normally distributed.

In contrast, a distribution shape-enforcing scale transformation is a transformation of the raw scores to scores with predefined mean, standard deviation, and shape. This transformation is a monotone, typically nonlinear, transformation. Examples are the percentile and decile transformations, which enforce a uniform distribution, the stanine transformation, which enforces a (discretized) normal distribution, and normalized *Z*-/*T*-/IQ-/Wechsler-score transformations, which enforce a normal distribution.

In choosing which type of norm-referenced scores to use for a test, it is important to consider the possible and required scales of measurement and the number of possible scores. First, the scale of measurement of norm-referenced scores is ordinal, unless it can be motivated why they would be at the interval level. That is, raw scores are minimally at an ordinal level (otherwise a norm-referenced interpretation does not make sense), and a transformation cannot increase the measurement level. This implies that for distribution shape-invariant transformed scores, the scale of measurement is at the interval level only if the raw scores were at the interval level. For distribution shape-enforcing scale transformations, it seems difficult to justify an interval level, apart from rather specific cases. For example, the raw scores are at the interval level and the population raw scores are (approximately) distributed as is enforced, or norm-referenced scores comply with a specific item response theory (IRT) model. If an ordinal level is sufficient for the interpretation, then both transformations are fine. If an interval level is possible and desired, then one needs to use the type of desired transformation that allows for an interval level.

Second, the number of possible scores of norm-referenced scores is equal to or smaller than that of the raw scores. Percentiles and (normalized) *Z*-/*T*-/IQ-/Wechsler-scores offer the impression of (approximately) continuous scores. Therefore, they are warranted if the score range of the raw scores is substantial (say, as a rule of thumb, more than 40), or if a warning to the limited score range is provided, and one applies a continuity correction when transforming the raw scores to the standardized scores. If the score range is limited for the raw scores, one could opt for less fine-grained scales, such as deciles or stanines. In practice, multiple options could be appropriate. For example, if the raw scores would be on an ordinal level, and the range of scores would be substantial, then from a psychometric point of view, percentiles and (normalized) *Z*-/*T*-/IQ-/Wechsler-scores are equivalent choices. In that case, the presumed ease of interpretation for the user should be guiding, for example, staying in line with what is commonly used in other tests in the field.

##### Calculating the Norms

In settling the norms, one derives a norm-referenced score for each possible raw test score, for each reference population. To get distribution-preserving norm-referenced scores (e.g., a *Z*-score, calculated as a linear transformation of the raw scores), one needs to estimate the mean and standard deviation in the reference population. In case of a single or very few reference populations (e.g., females/males), one could estimate these per reference population. In case of more than a few reference populations (e.g., for each age, within the age range of the test), it is more precise and efficient to model the mean and standard deviation as a function of the norm predictors using regression and use these estimates to calculate the norm-referenced scores.

To get distribution shape-enforcing scale transformations (e.g., percentile-based scores or normalized *Z*-scores), one needs the distribution of the raw scores in the reference population. In case of a single, or very few reference populations, one could estimate these per reference population, using the available rank-based normalization methods, including the Blom, Rankit and Tukey methods (see for a comparison, Soloman & Sawilowsky, 2009). In case of more than a few reference populations (e.g., for each age, within the age range of the test), continuous norming is more precise and efficient. Different continuous norming approaches exist, which all involve regression modeling with the norm predictor(s), the raw scores, and the distributional information. These regression models are based on the notion that the relationships between the continuous predictor(s) (like age) and the distribution of the raw scores follow smooth functions—both in the direction of the predictor(s) (e.g., with increasing age the mean, median, 25th [1th, 2th, 3rd . . .] percentile of the raw scores increases gradually) and in the direction of the cumulative distribution function (e.g., for a given age, the probability of a score x or lower gradually increases with increasing x). For a review of continuous norming methods, we refer to Urban and colleagues (2024a).

Here, we explicitly mention the two continuous norming methods for which up to date software is available, cNORM (Lenhard et al., 2018) and GAMLSS-based norming (Voncken et al., 2019b). cNORM can be seen as distribution-free norming, in that no specific distributional assumptions are made on the distribution of the test scores. GAMLSS-based norming is parametric norming, in that it assumes that the raw score distribution (conditional upon the norm predictors) follows one of the generalized additive models for location, scale, and shape (GAMLSS; Rigby & Stasinopoulos, 2005; Stasinopoulos, 2017). GAMLSS allows for modeling many different types of (test score) distributions, implying that for many types of normative data, a good-fitting GAMLSS model can be found.

Tutorials are available for cNORM (Gary et al., 2023) and GAMLSS-based norming (Timmerman et al., 2021), both including guidance on model selection and what to report (e.g., assumption checks) (cf. Question A7a). Whatever method is used to norm the data, it is key to select a good-fitting model, because norms founded on an ill-fitting model will be incorrect, i.e., deviate substantially from the population norms. The model selection involves defining a series of candidate models (e.g., specify the candidate distributions) (cf. Question A7a, i), and how to relate the norm predictor(s) to the outcome variable (e.g., using polynomials up to order 5) (cf. Question A7a, ii), assessing the model fit via statistical measures (e.g., Bayesian Information Criterion [Schwarz, 1978]) and visual diagnostics (e.g., worm plot, centile plot as a function of the norm predictor(s)) (cf. Question A7a, v, vi).

In the tutorials for cNORM and GAMLSS-based norming, it is assumed that the normative sample is representative of the normative population. If the normative sample is clearly non-representative with respect to one or more background variables, one needs to use an adjustment method to achieve a good (i.e., unbiased) estimate of the test score distribution per reference population. If one has reliable information on the joint distribution of auxiliary variables (i.e., background variable(s) and norm predictor(s)), a good approach is GAMLSS-based norming with multilevel regression and poststratification (GAMLSS-MRP; De Vries et al., 2025), or weighted cNORM (Gary et al., 2023). In the absence of reliable information on the joint distribution of auxiliary variables, one can estimate this information based on the normative sample data and use multilevel regression with so-called raking (Bruch & Felderer, 2023) (cf. Question A7a, iii).

The methods discussed so far are all based on raw test scores. Recently, item response–based continuous norming has been advocated in those cases for which the model holds, because of its higher precision over raw test score–based methods (Heister et al., 2024).

#### Sample size and design (cf. Question A6c)

For methods to calculate the norms per reference population separately, guidance on a minimally required sample size is available as a rule of thumb. For important decisions, *N* ≥ 400 per reference population is considered as good, and 300 ≤ *N* ≤ 400 as sufficient (Evers et al., 2009, p. 23).

For the continuous norming methods, as a rule of thumb, moderately large representative samples per age group (e.g., *n* = 100 to 200) are recommended for most application cases (Lenhard et al., 2019). This recommendation is founded upon simulation results under random sampling for cNORM. This applies to GAMLSS-based norming as well, since the latter’s efficiency seems to be similar to cNORM in simulation studies (e.g., Heister et al., 2024).

For GAMLSS-based norming, a minimally required sample size and an optimal design (e.g., sample sizes per age group) can be actually calculated, using certain assumptions. Specifically, assuming normality of the residuals applied to data from a simple random sample, statistical methods are available to calculate the minimally required sample size and to set the sampling design (Innocenti et al., 2023; Oosterhuis et al., 2016). Yet, in parametric norming practice, typically data result from quota sampling rather than random sampling and one needs a more complicated regression model that allows for non-normal distributions (Timmerman et al., 2021; Urban et al., 2024a). This means that the currently available guidance is generally too limited to settle the sample size for a normative study. To determine the minimally required sample size and design based on the available guidance for ordinary regression, one could use the presumed model that seems closest to the expected regression model for the normative data to be collected. This sample size can then be used as a lower boundary to what is needed.

For all continuous norming methods, it holds that when a nonlinear effect of a continuous norm predictor (e.g., age) is to be expected, it is wise to collect more data at the boundaries of this norm predictor, if the test is still suitable for the individuals concerned (Timmerman et al., 2021). A relatively larger sample size at the boundaries can remedy the limited precision at the boundaries in nonlinear regression models. Finally, if an inflection point is to be expected, then it is wise to collect more data in the region of the norm predictor where the inflection point is expected, in order to estimate the inflection point with sufficient precision.

#### Data collection

The data collection among the sampled individuals should take place under the standardized conditions as prescribed for the test under study (cf. Question A6e). For example, if the test would be released as a digital test, the norms must be based on scores gathered with this digital test, and not with a paper-pencil test. The data to be collected include the scores on the individual items of the test, on the norm predictors, and on the background variables, and the date of testing. The latter is important because changes in the status of a population on the trait measured may change the norms of a test measuring that trait (Hogan & Tsushima, 2016, p. 51). Perhaps the best-known example is the “Flynn effect,” the finding that the measured intelligence scores increase across successive generations (Flynn, 2011). Because all test norms may become outdated, it is needed to review the need for updating norms after some time (cf. Question A6f). The required timing depends on the test content and intended use(s) (American Educational Research Association et al., 2014, p. 84). In review systems for evaluating test quality, norms have been qualified as outdated when no new norming has been performed in the last 8 years (Diagnostik-Und Testkuratorium, 2018) or 20 years (EFPA, 2013; Evers et al., 2009).

##### Variations in Terminology Used in Step 3A

The terms auxiliary variable, norm predictor, and background variable were not used in any source included in our literature review. We decided to adopt the terms because in a norming context it is important, on one hand, to explicitly distinguish norm predictors from background variables, and on the other hand, to have a term for the joint of these variables; the term auxiliary variable is well-known in survey methodology (e.g., Valliant et al., 2018, p. 1). Authors who discussed these variables, without making a distinction with respect to their status in norming, denote these as demographic variables (Heffelfinger, 2014; Wasserman & Bracken, 2012) or client variables (Vacha-Haase, 2013).

Rather than using the term norm predictor, authors used the term explanatory variable (Lenhard et al., 2019), or a description pointing at the different norms for each reference population, as “separate ethnic group norms” (Cagigas & Manly, 2014), “subgroup norms” (Groth-Marnat & Wright, 2016a), “age-reference norms” (Holdnack, 2019), “gender-specific norms versus combined norms” (Butcher et al., 2013), and “gender-based norms versus non-gendered norms” (Brabender & Mihura, 2016). Others used descriptions as “similar background characteristics (e.g., education level)” (Holdnack, 2019), “normative data (corrected for age, education, gender, and race/ethnicity),” and “demographically corrected normative data” (Mitrushina et al., 2005).

#### Step 4A. Construct the Norms Based on the Normative Sample Data

For all raw scores, the norms entail their accompanying norm-referenced score. Once the norms are settled for each reference population, using the method chosen under Step 3A, it should be decided how to make them available to the user. For example, if age is a norm predictor, norms are settled for each possible age within the age range of the test’s target population. These norms can be provided via computer scoring, allowing to show the norms at any specific age, and/or in norm tables for consecutive (small) age intervals. The widths of the age intervals must be chosen such that per raw score the norm-referenced scores for consecutive age intervals are close to each other (cf. Question A7b). Typically, there is a gradual change in norms across age, meaning that the same raw score yields a gradually descending norm-referenced score with increasing age. Therefore, abrupt changes between age intervals would occur when age intervals are too broad in setting up the norm tables. In the case of computer scoring, we advise to offer the user the opportunity to inspect the norms themselves, in plots and/or tables. This allows the user to see any particularities in the norms, like floor and ceiling effects, and large jumps in norm-referenced scores associated with two adjacent raw scores (due to a limited score range).

To explicitly alert the test user to the imprecision inherent to an observed test score, it is good practice to express the degree of measurement error via a confidence interval (e.g., 95%; Smith Harvey, 2013, p. 46) and thus account for the reliability in the reference population (American Educational Research Association et al., 2014, p. 45; cf. Question A8). This can be done by calculating the desired confidence interval for the raw scores based on the group model from classical test theory (Charter & Feldt, 2001). By applying the norms to the boundaries of these raw score intervals, one can obtain the boundaries of the normed scores. Note that this interval does not account for uncertainty due to sampling fluctuations in the estimated norms. In practice, this source of imprecision is typically ignored, although a method exists to express the uncertainty due to norming (Voncken et al., 2019a).

Finally, the interpretation of a norm score and its associated confidence interval needs to be described as precisely and explicitly as possible, thereby providing a realistic view on their interpretational value (cf. Question A9). As an example, one may read about the interpretational value of IQ-scores (Ruiter et al., 2017; Tellegen, 2004; Westhoff & Kluck, 2014, p. 77).

### For Criterion-Referenced Scores

#### Step 2B. Select the Approach to Set the Cut Scores and the Required Decisions

A criterion-referenced test score expresses the performance achieved in comparison to a specified performance, without making direct reference to the performance of others. A criterion-referenced test score can typically be interpreted in terms of a likelihood. In the context of psychological tests, the likelihood may be, for example, on the presence of some psychopathology or on a successful job performance (American Educational Research Association et al., 2014, p. 96). In the context of an educational setting, it may be, for example, on responding correctly to certain items measuring a particular type of proficiency. We leave aside the latter context, thereby excluding credentialing tests (see Cizek et al., 2016), although they are usefully applied in a human resource practice and personnel selection.

For psychological tests, criterion-referenced scores are, as far as we can judge, expressed in terms of a grouping. This is done by identifying cut point(s) for raw test scores. The raw test scores are typically an unweighted sum score of individual items belonging to the scale involved. Three approaches are available to setting the cut scores, namely an empirically based approach, a norm-referenced approach, and an approach based on the judgment of experts in the field. As far as we could trace, only the first two are used for psychological tests (cf. Question B4).

First, an empirically based approach to derive cut scores is the optimal approach (cf. Question B5a). This means that the cut scores are based on their predictive efficacy of a relevant criterion (Frick & Cornell, 2003, p. 269). To this end, one would need data on both the test’s raw scores and a relevant criterion. Popular classification accuracy statistics are the sensitivity and specificity, and the positive predictive value and negative predictive value (Hunsley & Allan, 2019, p. 14). Because these classification accuracy statistics of a given cut score strongly depend on the base rate of the criterion involved, it is needed to consider the value of a cut score in a wide variety of settings and populations (Frick & Cornell, 2003, p. 271), relevant to the aim of the test’s use(s). Good examples of such evaluations among a wide range of populations are the Hospital Anxiety and Depression Scale (HADS; Zigmond & Snaith, 1983), as examined by Bjelland and colleagues (2002), and the Patient Health Questionnaire (PHQ-9; Kroenke et al., 2001), as examined by Costantini and colleagues (2021). A receiver operating characteristic (receiver operating characteristic curve (ROC)) depicts the sensitivity and specificity across a potential range of cut scores (Hunsley & Allan, 2019, p. 15). Therefore, it can be a useful aid to identify the cut score that optimally balances the test’s sensitivity and specificity, given the aim(s) of the test. Because the test may be used for different purposes, it can be good to report a full table with cut scores for a range of combinations of sensitivity and specificity. Furthermore, because the relationship between the test scores and the criterion may depend on auxiliary variable(s), like age, one may need different cut scores for different reference populations (Hunsley & Allan, 2019, p. 14).

To set these empirically based cut score(s) in standardization practice (cf. Question B6a), one needs to determine the criterion to be used to settle the cut scores for each reference population, possibly for a range of combinations of sensitivities and specificities. Furthermore, one needs to specify the procedure to determine the cut score (e.g., based on a prespecified balance between the sensitivity and specificity, and/or the positive predictive value and negative predictive value of the cut scores in a given reference population).

It is also possible to derive empirically based ordinal cut scores. This is applied in educational testing, like in the National Assessment of Educational Progress (NAEP; Beaton & Allen, 1992) and the Program for International Student Assessment (PISA; OECD, 2024). The derivation of ordinal cut scores is based on the assumption that individuals at a specific proficiency level have a high probability (e.g., above 60%) of correctly solving items at that proficiency level and below. IRT-based modeling allows for calculating the probability of solving an item correctly given its difficulty and the individual’s proficiency level. Thus, IRT enables to determine the proficiency level required to have a high probability of solving items of a specific difficulty correctly. This, however, requires items to be assigned to specific proficiency levels.

There are three different methods to assign items to proficiency levels (Harsch & Hartig, 2011). The first method, known as scale anchoring, assigns items to proficiency levels post hoc using predefined cut-offs (Beaton & Allen, 1992). Based on the assigned items, the proficiency levels are then described. This method, however, may not lead to clearly interpretable cut score(s) and proficiency levels. The second method predicts item difficulties a priori using item characteristics, for example, through the Linear Logistic Test Model or the Least Square Distance Model (Romero & Ordonez, 2014). The predicted item difficulties can then be used to establish the cut scores. The interpretation of the resulting proficiency levels is derived from the respective item characteristics. The third method starts with predefined proficiency levels and uses items that are intended to represent these levels. By estimating the item difficulties and the respective probability of solving them given the latent trait, cut scores between the predefined proficiency levels can be established (OECD, 2024).

Second, the cut scores are sometimes based on norm-referenced scores (Hogan & Tsushima, 2016, p. 51) from a reference population relevant to the aim of the test’s use (cf. Question B5b). This implies that multiple cut scores may be needed, for various reference populations, and possibly also for various aims of the test (Hunsley & Allan, 2019, p. 14). For example, for the SDQ (Goodman, 1997), the cut scores are typically determined such that the 10% most extreme scoring individuals in the total reference population are classified as “abnormal,” the 10% next-to-most-extreme scoring individuals as “borderline,” and the remaining 80% as “normal” (Vugteveen et al., 2022). As discussed under Step 2A, a reasonable choice for the reference population seems to let it depend on age, and possibly on gender as well.

To set cut score(s) based on norm-referenced scores in standardization practice (cf. Question B6b), one needs to define the test’s reference population(s) and the test’s normative population. Furthermore, one needs to select the percentile(s) for the cut score(s), which must be reasonable given the reliability of the raw test scores and the test aim. The latter needs to be substantiated, for example, based on a golden standard, or prevalence statistics, and statistics required according to a settled protocol or standard. When the cut scores cannot be substantiated, the resulting classification does not have a criterion-referenced interpretation. For example, IQ classifications (e.g., Flynn, 2011; Ruiter et al., 2017; Sattler, 2008) do not relate to a criterion, and thus only have a norm-referenced interpretation.

Third, the cut scores may be based on the judgment of experts in the field (Hogan & Tsushima, 2016, p. 51). To this end, various judgmental procedures have been proposed, denoted as standard-setting procedures (Cizek & Bunch, 2007), stemming from an educational setting. For example, results of exams of the New York State are categorized based on standard-setting procedures (New York State Education Department, n.d.). We could not find any example of a psychological test for which cut scores were derived using a standard-setting procedure. Therefore, we do not discuss this approach further here.

#### Step 3B. Design and Carry Out the Study to Gather the Required Data

For empirically based cut score(s), one needs to decide upon the criterion (or criteria)—to be used as the gold standard(s)—and the method to determine the cut score(s) based on the test scores. Furthermore, it should be decided which are the relevant population(s) to sample from (cf. Question B6a). The sampling procedure can be determined along the lines discussed in Step 3A for the norm-referenced scores. Ideally, one would also collect data among second, independent sample(s) from the relevant population(s), to (cross-)validate the cut score(s) identified. For example, the cut scores presented with the HADS (Zigmond & Snaith, 1983) were determined based on data from 98 patients from general medical outpatient clinics. In 24 subsequent studies, samples from a range of patient populations (e.g., primary care, cancer, internal medicine) and a community population were assessed, resulting in various cut scores. It is essential to precisely define the relevant population(s) (Bjelland et al., 2002).

For cut score(s) based on norm-referenced scores, one needs to design the study as described in Step 3A for the norm-referenced scores (cf. Question B6b). Ideally, also here, one would have a gold standard, that is, define a criterion in line with the aim of the test and decide upon a good measure for that criterion. This allows for assessing the accuracy of the cut score(s) identified, and thus to validate the cut score(s).

#### Step 4B Based on the Empirical Data, Determine the Cut Score(s), Validate the Cut Scores, and Describe the Interpretation

For empirically based cut score(s), follow the specified procedure to determine the cut score(s) (e.g., based on a prespecified balance between the sensitivity and specificity, or the positive predictive value and negative predictive value of the cut scores, or for a range of combinations of sensitivity and specificity).

For cut score(s) based on norm-referenced scores, one needs to settle the norms as described in Step 4A. Subsequently, determine the cut score(s) associated with the settled percentile(s) for the cut score(s), for each reference population of interest.

Irrespective of the approach taken to determine the cut score(s), the validation of the cut scores needs to be done based on new sample(s) from the relevant population(s), which were not used to compute the cut scores. Appropriate accuracy measures should be used, such as the sensitivity and specificity, and/or the positive predictive value and negative predictive value.

Finally, the interpretation of the categories resulting from applying the cut scores needs to be described, for all relevant populations, as precisely and explicitly as possible.

#### Step 5. Reporting

The report in a test manual should cover the construction of the standardized test scores, as well as instructions to the test users for clear and useful score reports.

The reporting on the construction of standardized test scores should be such that it is transparent what has been done, and how these choices are motivated and underpinned (cf. Question 10). Steps 1 to 4 discussed above can provide guidance for what to describe. The information can all be included in the technical manual of a test, or parts can be described in the back-end documentation of the test, which can be made available if needed, for example, to test reviewers. The latter can be done, for example, because otherwise the technical manual becomes needlessly long and difficult to access, or because commercial interests preclude an open reporting of information. Furthermore, it is advised to make available (e.g., on OSF [see Note 1]) analyses scripts and, if possible, anonymized data. The latter can be precluded for commercial reasons, because norm data themselves are not copyright protected.

The key purpose of score reporting of a psychological test is to convey its interpretation to intended users. A clear and useful score report thus supports users in making appropriate score inferences (Hambleton & Zenisky, 2013). Therefore, it needs to be included in the user’s manual (cf. Question 11). To guide a report development, we refer to Hambleton and Zenisky (2013, pp. 479–494), who provide an overview of reporting options and considerations and introduce a formal process for report development, including a review sheet. For advice on a broad range of issues on written communication of test results, including, for example, readability, report length, computer scoring, and computer generated reports, we refer to Smith Harvey (2013, pp. 37–42). We advice to also include exemplary case reports including completed score forms.

We recommend to send out a draft manual to a selection of potential test users, to ask for feedback on the clarity, completeness, and practical usefulness of the manual (cf. Question 12).

## How to Use the GRoNC

The primary audience we had in mind when developing the GRoNC was test constructors and test reviewers. Yet, the overview of key aspects relevant in norming a psychological test is of use for anyone involved in psychological testing, in research, and in (clinical) practice. Moreover, careful norming impacts all individuals who are assessed with psychological test, like for screening, referral or selecting targeted interventions. Test constructors may refer to the GRoNC early during standardization study conceptualization and design, because knowledge of the guidelines and the theoretical background can aid in shaping the study design. Furthermore, the GRoNC can be of help in the writing process of the test manual, to cover all relevant aspects—providing the technical justification as well as providing guidance for test users in interpreting the standardized scores. Information on these aspects needs to be provided in a manual accompanying the test, and possibly Supplementary digital materials in a repository (e.g., OSF, PsyArXiv). Editors can use the GRoNC when providing authors with feedback on their test manuals. Test reviewers can refer to the GRoNC when reviewing standardized tests. Local bodies that review standardized tests based on a standardized review scheme may use the GRoNC to update their review scheme with respect to standardization.

We recommend all users to apply the GRoNC with professional judgment. That is, for each item, consider to what extent it is relevant for the study at hand, and to what extent it can be realistically met, thereby refraining from simply ticking-the-boxes. Make sure that all items are covered in the test documentation—also those that are not met, including a motivation. The GRoNC is meant to stimulate test constructors to achieve a high-quality standardization—given the practical constraints that are inevitable. For example, from a certain clinical population, it can be impossible to recruit a large sample. In that case, it can be a better choice to include standardized scores based on a small sample, than to refrain from providing standardized scores related to this clinical population at all. Obviously, it is key that the test constructor then alerts the reader to the consequences (i.e., uncertainty in the standardized scores) and its effects on the interpretability.

## Conclusion

This article has introduced guidelines for the development of and reporting on norm-referenced and criterion-referenced scores, standardized scores for short. The recommended elements that should ideally be considered and presented have been discussed in detail, based on a series of questions. The guidelines also provide a theoretical foundation for developing standardized scores. This provides a basis for further (psychometric and statistical) developments of standardization methods.

We expect an improvement in the development of and reporting on standardized scores. The potential for this is demonstrated by studies of existing reporting guidelines. For example, the use of Consolidated Standards of Reporting Trials (CONSORT) checklists is associated with an improved reporting of randomized controlled trials (Plint et al., 2006; Turner et al., 2012). We expect a harmonization of the review process for standardized tests and an increase in awareness among test developers and test users on the quality of the standardized scores. The implementation of the guideline is contingent on its publicity and whether organizations involved in the assessment of tests require the use of the guideline. We will disseminate the GRoNC by publishing the GRoNC in the EQUATOR network and inform organizations involved in the assessment of tests and test publishers via email, asking to use the GRoNC. Herewith, we will explicitly solicit for their evaluation of the reporting guideline. By doing this, we hope to gain information and insights on the use of the GRoNC. We plan to evaluate the GRoNC’s impact after 3 years of publication, in a study among test developers who applied GRoNC. If needed, the GRoNC can be adapted and updated based on new developments in standardized test score methodology.

## Supplemental Material

sj-docx-1-asm-10.1177_10731911251371395 – Supplemental material for The GRoNC: Guidelines for Reporting on Norm-Referenced and Criterion-Referenced Scores Supplemental material, sj-docx-1-asm-10.1177_10731911251371395 for The GRoNC: Guidelines for Reporting on Norm-Referenced and Criterion-Referenced Scores by Marieke E. Timmerman, Annelies De Bildt and Julian Urban in Assessment

## Notes

1. https://osf.io/w6uqp/.
