# Supplementary material for: The GRoNC: Guidelines for Reporting on Norm-Referenced and Criterion-Referenced Scores
Source: Assessment. 2025 Sep 24;33(6):954–72. doi: 10.1177/10731911251371395 (PMC13379607; doi:10.1177/10731911251371395)
Supplement: sj-docx-1-asm-10.1177_10731911251371395 – Supplemental material for The GRoNC: Guidelines for Reporting on Norm-Referenced and Criterion-Referenced Scores [file sj-docx-1-asm-10.1177_10731911251371395.docx]

# Supplementary material for the GRoNC-Checklist: Guidelines for Reporting on Norm-referenced and Criterion-referenced scores

## Appendix A: Systematic approach to creating the GRoNC

The GRoNC has been developed following the systematic approach for the creation of health reporting guidelines (Moher et al., 2014), as advocated by the EQUATOR Network (EQUATOR Network, n.d.), as far as applicable for our purposes. This systematic approach is summarized in a checklist (Moher et al., 2010). In Table A1, we present this checklist in columns one and two, and indicate whether and how we applied each item, including a rationale, in column three.

Table A1. EQUATOR Checklist and its application in creating the GRoNC

| EQUATOR Checklist | | Application in creating GRoNC (as described in this section in the paper) |
| --- | --- | --- |
| Step | Item number - Detail. |  |
| Initial steps | 1. Identify the need for a guideline  1.1. Develop new guidance  1.2. Extend existing guidance  1.3. Implement existing guidance | Yes; via the systematic literature study, it was determined that there was a need for a guideline, and that it was most appropriate to develop new guidance, rather than extend or implement existing guidance (A. Literature review; details in Appendix B). |
|  | 2. Review the literature  2.1. Identify previous relevant guidance  2.2. Seek relevant evidence on the quality of reporting in published research articles  2.3. Identify key information on the potential sources of bias, and other deficiencies, in such study reports | Yes; via the systematic literature study, previous guidance was identified, and evidence on the quality of reporting in published manuals (rather than research articles, given the scope of our guideline), and sources of bias and other deficiencies were sought (A. Literature review; details in Appendix B). |
|  | 3. Obtain funding for the guideline initiative | Yes; the work is supported by a grant to the first author (Funding statement). |
| Premeeting activities | 4. Identify participants | Yes; we identified 14 theoretical experts, and 20 test developers to invite (C. Consulting experts; details in Appendix C). |
|  | 5. Conduct a Delphi exercise | Yes, using a ‘reactive’ Delphi method (McKenna, 1994), where participants respond to a previously constructed version of items; These were constructed by the authors. |
|  | 6. Generate a list of items for consideration at the face-to-face  meeting | Yes; we developed GRoNC, version 1, consisting of questions and explanations, for consideration by the participants. The GRoNC consists of questions and explanations, which needs careful reading to fully process.  Given this nature of the GRoNC, we deemed it more appropriate to ask for written feedback, rather than having a face-to-face meeting (B. Generate GRoNC, version 1). |
|  | 7^a^. Prepare for the face-to-face meeting  7.1. Decide size and duration of the face-to-face meeting  7.2. Develop meeting logistics  7.3. Develop meeting agenda  7.3.1. Consider presentations on relevant background topics, including summary of evidence  7.3.2. Plan to share results of Delphi exercise, if done  7.3.3. Invite session chairs  7.4. Prepare materials to be sent to participants prior to meeting  7.5. Arrange to record the meeting  8^a^. Present and discuss results of premeeting activities and relevant evidence  8.1^a^. Discuss the rationale for including items in the checklist | Yes, in an alternative form; given the nature of the GRoNC, we asked for written feedback, in two rounds, with first theoretical experts and second test constructors (B. Generate GRoNC, version 1; details in Appendix C). |
|  | 8.2. Discuss the development of a flow diagram  8.3^a^. Discuss strategy for producing documents; identify who will be involved in which activities; discuss authorship  8.4^a^. Discuss knowledge translation strategy | Yes; the author team discussed the potential use of a flow diagram, the strategy for producing documents etcetera, and the knowledge translation strategy. |
| Postmeeting activities | 9^a^. Develop the guidance statement | Yes; the author team developed the GRoNC (B. Generate GRoNC, version 1; C. Consulting experts; details in Appendix B and C). |
|  | 9.1. Pilot test the checklist | No; given that extensive feedback has been provided by theoretical experts and test developers, we feel it would not be needed to additionally pilot test the GRoNC. |
|  | 10. Develop an explanatory document (E&E) | Yes; that is, the GRoNC consists of questions and explanations, and thus already includes an explanatory document. |
|  | 11. Develop a publication strategy  11.1. Discuss concurrent simultaneous publications with editors | Yes; the author team developed a publication strategy (submission to Journal), and identified that there are no concurrent simultaneous publications. |
| Postpublication activities | 12^a^. Seek and deal with feedback and criticism | Yes; the authors will actively present the GRoNC to relevant bodies and be open for, respond to and act upon feedback. |
|  | 13 ^a^. Encourage guideline endorsement | Yes; the authors formulated a dissemination strategy, including sending the GRoNC to relevant organizations, test publishers and test developers. |
|  | 14. Support adherence to the guideline | Yes; the authors will be open to support adherence. |
|  | 15. Evaluate the impact of the reporting guidance | Yes; the authors plan to evaluate the impact after 3 years of publication. |
|  | 16. Develop website | No; the authors deem the publication in a journal as sufficient. |
|  | 17. Translate guideline | No; since the GRoNC is in the common scientific language English, the authors see no need to translate it. |
|  | 18. Update guideline | Yes; the authors will be open to update the GRoNC whenever it appears to be needed. |

^a^ Core set of items – see text (Moher et al., 2010)

## Appendix B: Stage 1. Step 1. Literature review

We used EbscoHost Search Syntax for PsychInfo Literature Searches to 1) identify what need there may be for new guidelines and 2) review the literature on standardized test scores construction and interpretation.

A summary of the process of our systematic literature search is provided in our flow diagram in Figure A1.


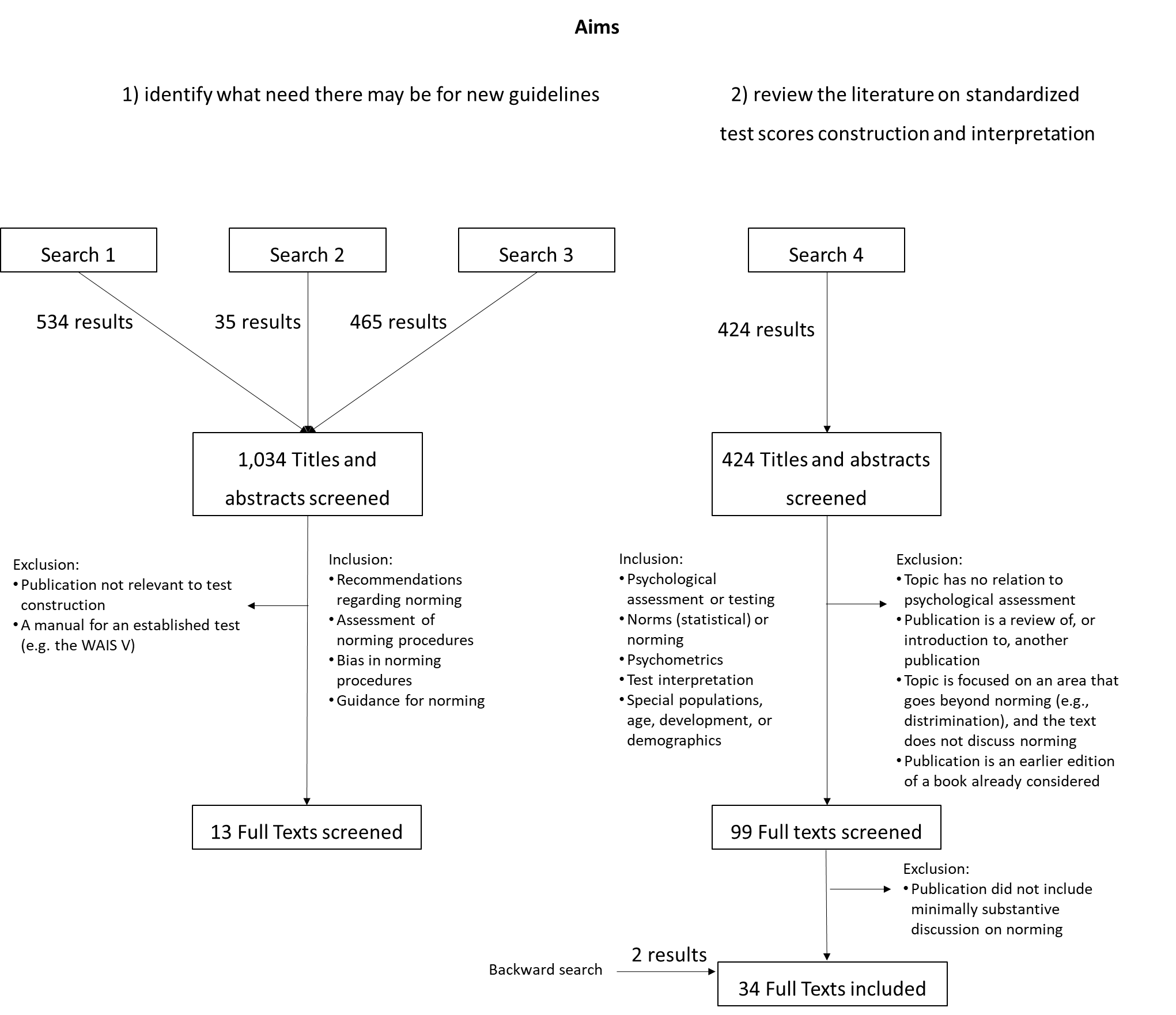


Figure A1. Flow diagram showing the process of our systematic literature search. Note that we aimed to conduct a systematic literature search and not a systematic review.

### Syntaxes

To 1) identify what need there may be for new guidelines, we involved search terms related to testing, guidelines, construction and norming. We used three searches to adequately span the literature, while keeping the number of results manageable.

***Search 1:*** Books, articles published in academic journals, and electronic collections, in English, from 1998, carried out on 26th April, 2023.

(TI *test*)* OR (AB *test*)* OR (TI *scale*)* OR (AB *scale*)* OR (TI *instrument*)* OR (AB *instrument*) OR (TI questionnaire*) OR (AB questionnaire*)*

AND

(TI *guide*)* OR (AB *guide*)* OR (TI *checklist*)* OR (AB *checklist*)* OR (TI *manual*)* OR (AB *manual*)* OR (TI *flow diagram*)* OR (AB *flow diagram*)* OR (TI *structured text)* OR (AB *structured text*)

AND

(TI construction) OR (AB construction) OR (TI constructor*) OR (AB constructor*)

*534 results*

***Search 2*:** Books, articles published in academic journals, and electronic collections, in English, from 1998, carried out on 26th April, 2023.

(TI *test*)* OR (AB *test*)* OR (TI *scale*)* OR (AB *scale*)* OR (TI *instrument*)* OR (AB *instrument*) OR (TI questionnaire*) OR (AB questionnaire*)*

*AND*

(TI *guide*)* OR (AB *guide*)* OR (TI *checklist*)* OR (AB *checklist*)* OR (TI *manual*)* OR (AB *manual*)* OR (TI *flow diagram*)* OR (AB *flow diagram*)* OR (TI *structured text)* OR (AB *structured text*) AND (TI *norming)* OR (AB *norming)*

*35 results*

***Search 3:*** Books, articles published in academic journals, and electronic collections, in English, from 2013. Carried out on 26th April, 2023

(TI *test*)* OR (AB *test*)* OR (TI *scale*)* OR (AB *scale*)* OR (TI *instrument*)* OR (AB *instrument*) OR (TI questionnaire*) OR (AB questionnaire*)*

AND

(TI *guide*)* OR (AB *guide*)* OR (TI *checklist*)* OR (AB *checklist*)* OR (TI *manual*)* OR (AB *manual*)* OR (TI *flow diagram*)* OR (AB *flow diagram*)* OR (TI *structured text)* OR (AB *structured text*)

AND

(TI *norm)* OR (AB *norm)*

*465 results*

To 2) review the literature on standardized test scores construction and interpretation, we used search terms related to handbook, psychological assessment, neuropsychology, clinical psychology, special needs education and human resource management.

***Search 4:*** Books, peer reviewed articles, and electronic collections (no dissertations), in English, from 1998, carried out on 11 May, 2023.

(TI *Handbook)* OR (AB *Handbook)* OR (TI *compendium)* OR (AB *compendium)*

AND

(TI *“psychological assessment”)* OR (AB *“psychological assessment”)* OR(TI *neuropsychology)* OR (AB *neuropsychology)* OR (TI *“clinical psychology”)* OR (AB *“clinical psychology”)* OR (TI *“special needs education”)* OR (AB *“special needs education”)* OR (TI *“human resources management”)* OR (AB *“human resources management”*)

*424 Results*

### Inclusion and Exclusion Criteria for the title and abstract screening, applied by [details omitted for double-anonymized peer review].

To 1) identify what need there may be for new guidelines, we applied the following criteria.

Inclusion criteria were any mention of:

- Recommendations regarding norming
- Assessment of norming procedures
- Bias in norming procedures
- Guidance for norming

Exclusion criteria screening were:

- Publication not relevant to test construction
- A manual for an established test (e.g. the WAIS V)

To 2) review the literature on standardized test scores construction and interpretation, we applied the following criteria.

Inclusion criteria were any mention of:

- Psychological assessment or testing
- Norms (statistical) or norming
- Psychometrics
- Test interpretation
- Special populations, age, development, or demographics

Exclusion criteria were:

- Topic has no relation to psychological assessment
- Publication is a review of, or introduction to, another publication
- Topic is focused on an area to which norming is irrelevant, i.e., discrimination, stereotyping, morality
- Publication is an earlier edition of a book already considered

### Inclusion and Exclusion Criteria for the full text screening, applied by [details omitted for double-anonymized peer review].

To 1) identify what need there may be for new guidelines, we applied the following inclusion criterion.

- Publication did include recommendations for reporting on the construction and interpretation of standardized test scores

To 2) review the literature on standardized test scores construction and interpretation, we applied the following inclusion criterion.

- Publication did include a minimally substantive discussion on norming.

### Full texts retained

Ad 1) identify what need there may be for new guidelines

Lenhard et al., 2019

Ad 2) review the literature on standardized test scores construction and interpretation

Brabender, 2022; Brabender & Mihura, 2016; Butcher et al., 2013; Cagigas & Manly, 2014; Clinton & Olvera, 2014; Conant, 2014; Freedman & Manly, 2018; Freeman & Chen, 2019; Frick & Cornell, 2003; Goldstein et al., 2019; Groth-Marnat & Wright, 2016a, 2016b; Hale et al., 2016; Hambleton & Zenisky, 2013; Hays, 2013; Heffelfinger, 2014; Hogan & Tsushima, 2016; Holdnack, 2019; Horn et al., 2013; Hunsley & Allan, 2019; Kolen & Hendrickson, 2013; Krishnamurthy, 2019; Little & Akin-Little, 2014; Mihura & Brabender, 2016; Mindt et al., 2019; Mitrushina et al., 2005; Paltzer, 2018; Pedraza, 2018; Pontón, 2001; Reynolds & Livingston, 2019; Reynolds & Mason, 2009; Rourke & Bartolini, 2016; Streiner, 2021; Suzuki et al., 2005; Vacha-Haase, 2013; Wasserman & Bracken, 2012; Weiler et al., 2019; Wright & Nickleberry, 2016

2 text retained via backward search: American Educational Research Association et al., 2014; Smith Harvey, 2013.

### References

American Educational Research Association, American Educational Research Association, & National Council on Measurement in Education (Eds.). (2014). *Standards for educational and psychological testing*. American Educational Research Association.

Brabender, V. M. (2022). Gender, gender identity, and sexual orientation in personality and personality assessment. In J. L. Mihura (Ed.), *The Oxford Handbook of Personality and Psychopathology Assessment* (2nd ed.). Oxford University Press. https://doi.org/10.1093/oxfordhb/9780190092689.013.28

Brabender, V. M., & Mihura, J. L. (2016). The construction of gender and sex, and their implications for psychological assessment. In *Handbook of Gender and Sexuality in Psychological Assessment* (pp. 3–43).

Butcher, J. N., Bubany, S., & Mason, S. N. (2013). Assessment of personality and psychopathology with self-report inventories. In K. F. Geisinger, B. A. Bracken, J. F. Carlson, J.-I. C. Hansen, N. R. Kuncel, S. P. Reise, & M. C. Rodriguez (Eds.), *APA handbook of testing and assessment in psychology: Testing and assessment in clinical and counseling psychology.* (pp. 171–192). American Psychological Association. https://doi.org/10.1037/14048-011

Cagigas, X. E., & Manly, J. J. (2014). Cultural neuropsychology: The new norm. In *Clinical Neuropsychology: A Pocket Handbook for Assessment* (3rd ed., Vol. 27, pp. 132–156). American Psychological Association. https://journals.lww.com/00146965-201412000-00008

Clinton, & Olvera, P. (2014). Norm-referenced assessment and bilingual populations. In S. Little & A. Akin-Little (Eds.), *Academic Assessment and Intervention* (0 ed.). Routledge. https://doi.org/10.4324/9780203108451

Conant, L. L. (2014). Neuropsychological assessment of developmental disorders: Learning disabilities, attention-deficit/hyperactivity disorder, autism spectrum disorders. In *Clinical Neuropsychology: A Pocket Handbook for Assessment* (3rd ed., pp. 527–551). American Psychological Association. https://journals.lww.com/00146965-201412000-00008

EQUATOR Network. (n.d.). Retrieved August 15, 2023, from https://www.equator-network.org/

Freedman, D., & Manly, J. J. (2018). Assessment of cognition in African American older adults. In G. E. Smith (Ed.), *APA Handbook of Dementia.* (pp. 107–123). American Psychological Association. https://doi.org/10.1037/0000076-006

Freeman, A. J., & Chen, Y.-L. (2019). Interpreting pediatric intelligence tests: A framework from evidence-based medicine. In *Handbook of Psychological Assessment* (pp. 65–101). Elsevier. https://doi.org/10.1016/B978-0-12-802203-0.00003-1

Frick, P. J., & Cornell, A. H. (2003). Child and adolescent assessment and diagnosis research. In M. C. Roberts & S. S. Ilardi (Eds.), *Handbook of Research Methods in Clinical Psychology* (pp. 262–283). Blackwell Publishing Ltd. https://doi.org/10.1002/9780470756980.ch13

Goldstein, G., Allen, D. N., & DeLuca, J. (2019). Adult comprehensive neuropsychological assessment. In *Handbook of Psychological Assessment* (pp. 227–273). Academic Press. https://doi.org/10.1016/B978-0-12-802203-0.00008-0

Groth-Marnat, G., & Wright, A. J. (2016a). The context of clinical assessment. In *Handbook of Psychological Assessment* (pp. 39–75). John Wiley & Sons, Inc.

Groth-Marnat, G., & Wright, A. J. (2016b). Wechsler memory scales. In *Handbook of Psychological Assessment* (pp. 215–242). John Wiley & Sons, Inc.

Hale, J. B., Wilcox, G., & Reddy, L. A. (2016). Neuropsychological assessment. In J. C. Norcross, G. R. VandenBos, D. K. Freedheim, & R. Krishnamurthy (Eds.), *APA Handbook of Clinical Psychology: Applications and Methods* (Vol. 3, pp. 139–165). American Psychological Association. https://doi.org/10.1037/14861-007

Hambleton, R. K., & Zenisky, A. L. (2013). Reporting test scores in more meaningful ways: A research-based approach to score report design. In K. F. Geisinger, B. A. Bracken, J. F. Carlson, J.-I. C. Hansen, N. R. Kuncel, S. P. Reise, & M. C. Rodriguez (Eds.), *APA Handbook of Testing and Assessment in Psychology: Testing and Assessment in School Psychology and Education.* (Vol. 3, pp. 479–494). American Psychological Association. https://doi.org/10.1037/14049-023

Hays, D. G. (2013). Understanding and transforming raw scores. In *Assessment in Counseling: A Guide to the Use of Psychological Assessment Procedures* (5th ed., pp. 105–120). American Counseling Association.

Heffelfinger, A. (2014). Issues in the assessment of children. In M. W. Parsons, T. A. Hammeke, & P. J. Snyder (Eds.), *Clinical Neuropsychology: A Pocket Handbook for Assessment* (3rd ed., pp. 114–131). American Psychological Association. https://doi.org/10.1037/14339-007

Hogan, T. P., & Tsushima, W. T. (2016). Psychometrics and testing. In J. C. Norcross, G. R. VandenBos, D. K. Freedheim, & R. Krishnamurthy (Eds.), *APA Handbook of Clinical Psychology: Applications and Methods* (Vol. 3, pp. 31–54). American Psychological Association. https://doi.org/10.1037/14861-003

Holdnack, J. A. (2019). The development, expansion, and future of the WAIS-IV as a cornerstone in comprehensive cognitive assessments. In *Handbook of Psychological Assessment* (pp. 103–139). Academic Press. https://doi.org/10.1016/B978-0-12-802203-0.00004-3

Horn, S. L., Mihura, J. L., & Meyer, G. J. (2013). Psychological assessment in adult mental health settings. In K. F. Geisinger, B. A. Bracken, J. F. Carlson, J.-I. C. Hansen, N. R. Kuncel, S. P. Reise, & M. C. Rodriguez (Eds.), *APA Handbook of Testing and Assessment in Psychology: Testing and Assessment in Clinical and Counseling Psychology* (Vol. 2, pp. 231–252). American Psychological Association. https://doi.org/10.1037/14048-000

Hunsley, J., & Allan, T. (2019). Psychometrics and psychological assessment. In M. Sellbom & J. A. Suhr (Eds.), *The Cambridge Handbook of Clinical Assessment and Diagnosis* (1st ed., pp. 9–24). Cambridge University Press. https://doi.org/10.1017/9781108235433.002

Kirk, C., & Vigeland, L. (2014). A psychometric review of norm-referenced tests used to assess phonological error patterns. *Language, Speech, and Hearing Services in Schools*, *45*(4), 365–377. https://doi.org/10.1044/2014_LSHSS-13-0053

Kolen, M. J., & Hendrickson, A. B. (2013). Scaling, norming and equating. In K. F. Geisinger, B. A. Bracken, J. F. Carlson, J.-I. C. Hansen, N. R. Kuncel, S. P. Reise, & M. C. Rodriguez (Eds.), *APA Handbook of Testing and Assessment in Psychology: Test Theory and Testing and Assessment in Industrial and Organizational Psychology* (Vol. 1, pp. 201–222). American Psychological Association. https://doi.org/10.1037/14047-000

Krishnamurthy, R. (2019). Gender considerations in self-report personality assessment interpretation. In *Handbook of Gender and Sexuality in Psychological Assessment* (pp. 128–148). Routledge.

Lenhard, A., Lenhard, W., & Gary, S. (2019). Continuous norming of psychometric tests: A simulation study of parametric and semi-parametric approaches. *PLOS ONE*, *14*(9), e0222279. https://doi.org/10.1371/journal.pone.0222279

Little, S., & Akin-Little, A. (2014). Methods of academic assessment. In S. Little & A. Akin-Little (Eds.), *Academic Assessment and Intervention* (0 ed.). Routledge. https://doi.org/10.4324/9780203108451

McKenna, H. P. (1994). The Delphi technique: A worthwhile research approach for nursing? *Journal of Advanced Nursing*, *19*(6), 1221–1225. https://doi.org/10.1111/j.1365-2648.1994.tb01207.x

Mihura, J. L., & Brabender, V. M. (2016). Sex, gender, and sexuality in psychological assessment: Where do we go from here? In *Handbook of Gender and Sexuality in Psychological Assessment* (pp. 681–708). Routledge.

Mindt, M. R., Arentoft, A., Coulehan, K., Summers, A. C., Tureson, K., Aghvinian, M., & Byrd, D. A. (2019). Neuropsychological evaluation of culturally/linguistically diverse older adults. In L. D. Ravdin & H. L. Katzen (Eds.), *Handbook on the Neuropsychology of Aging and Dementia* (pp. 25–48). Springer International Publishing. https://doi.org/10.1007/978-3-319-93497-6_3

Mitrushina, M., Boone, K. B., Razani, J., & D’Elia, L. F. (2005). Background. In *Handbook of Normative Data for Neuropsychological Assessment* (2nd ed.). Oxford University Press.

Moher, D., Schulz, K. F., Simera, I., & Altman, D. G. (2010). Guidance for developers of health research reporting guidelines. *PLoS Medicine*, *7*(2), e1000217. https://doi.org/10.1371/journal.pmed.1000217

Muñiz, J., & Bartram, D. (2007). Improving international tests and testing. *European Psychologist*, *12*(3), 206–219. https://doi.org/10.1027/1016-9040.12.3.206

Paltzer, J. Y. (2018). Assessment of age-related cognitive changes and dementia in Chinese and Chinese American older adults. In G. E. Smith & S. T. Farias (Eds.), *APA Handbook of Dementia.* (pp. 125–140). American Psychological Association. https://doi.org/10.1037/0000076-007

Pedraza, O. (2018). Neuropsychological assessment of Spanish-speaking older adults. In G. E. Smith & S. T. Farias (Eds.), *APA Handbook of Dementia.* (pp. 141–170). American Psychological Association. https://doi.org/10.1037/0000076-008

Pontón, M. O. (2001). Research and assessment issues with Hispanic populations. In M. O. Pontón & J. León-Carrión (Eds.), *Neuropsychology and the Hispanic patient: A clinical handbook.* Lawrence Erlbaum Associates Publishers.

Reynolds, C. R., & Livingston, R. (2019). How to develop an empirically based psychological test. In *Handbook of Psychological Assessment* (pp. 31–62). Elsevier. https://doi.org/10.1016/B978-0-12-802203-0.00002-X

Reynolds, C. R., & Mason, B. A. (2009). Measurement and statistical problems in neuropsychological assessment of children. In C. R. Reynolds & E. Fletcher-Janzen (Eds.), *Handbook of Clinical Child Neuropsychology* (pp. 203–230). Springer US. https://doi.org/10.1007/978-0-387-78867-8

Rourke, M. T., & Bartolini, E. (2016). A framework for considering gender in the cognitive assessment process. In *Handbook of Gender and Sexuality in Psychological Assessment* (pp. 108–127). Routledge.

Smith Harvey, V. (2013). Communicating Test Results. In *APA handbook of testing and assessment in psychology, Vol. 2. Testing and assessment in clinical and counseling psychology.* American Psychological Association.

Smits, N., Paap, M. C. S., & Böhnke, J. R. (2018). Some recommendations for developing multidimensional computerized adaptive tests for patient-reported outcomes. *Quality of Life Research*, *27*(4), 1055–1063. https://doi.org/10.1007/s11136-018-1821-8

Streiner, D. L. (2021). Test theory and measurement in assessment. In J. L. Mihura (Ed.), *The Oxford Handbook of Personality and Psychopathology Assessment* (2nd ed.). Oxford University Press. https://doi.org/10.1093/oxfordhb/9780190092689.013.7

Suzuki, L. A., Kugler, J. F., & Aguiar, L. J. (2005). Assessment practices in racial-cultural psychology. In R. T. Carter (Ed.), *Handbook of Racial-Cultural Psychology and Counseling: Training and Practice* (Vol. 2, pp. 297–315). John Wiley & Sons, Inc.

Vacha-Haase, T. (2013). Psychological assessment with older adults. In K. F. Geisinger, B. A. Bracken, J. F. Carlson, J.-I. C. Hansen, N. R. Kuncel, S. P. Reise, & M. C. Rodriguez (Eds.), *APA Handbook of Testing and Assessment in Psychology: Testing and Assessment in Clinical and Counseling Psychology.* (Vol. 2, pp. 555–568). American Psychological Association. https://doi.org/10.1037/14048-032

Wasserman, J. D., & Bracken, B. A. (2012). Fundamental psychometric considerations in assessment. In J. R. Graham, J. A. Naglieri, & I. B. Weiner (Eds.), *Handbook of Psychology* (2nd ed., Vol. 10, pp. 50–81). John Wiley & Sons, Inc. https://doi.org/10.1002/9781118133880.hop210003

Weiler, M. D., Willis, W. G., & Kennedy, M. L. (2019). Sources of error and meaning in the pediatric neuropsychological evaluation. In *Handbook of Psychological Assessment* (pp. 193–226). Elsevier. https://doi.org/10.1016/B978-0-12-802203-0.00007-9

Wright, A. J., & Nickleberry, L. (2016). Gender, sexuality, and assessment in adolescence. In *Handbook of Gender and Sexuality in Psychological Assessment* (pp. 552–577). Routledge.

## Appendix C: Stage 2.

### First round: theoretical experts

We asked theoretical experts for feedback on version 1 of the GRoNC-Checklist. In total, we approached 14 experts, via different sources, namely EFPA Board of assessment (<https://www.efpa.eu/working-groups/assessment>)[[1]](#footnote-1), the Dutch COTAN (<https://www.cotandocumentatie.nl/cotan/)1>, the German Diagnostik- Und Testkuratorium (<https://www.dgps.de/die-dgps/kooperationen/diagnostik-und-testkuratorium/>), and two authors of relevant and high-quality chapters from our literature review.

In February 2024, we sent out our invitation to participate, including the first version of the GRoNC and four questions, which are provided below. In total 11 experts provided their feedback, who are listed below. Eight of them answered the four questions explicitly, and six of them additionally provided detailed feedback, both related to content and text.

Author 1 carefully considered all feedback, and incorporated it in the text where deemed appropriate. From these responses, Author 1 extracted 10 points to discuss with Authors 2 and 3. Author 1 rewrote GRoNC-V0 based on the discussion, with contributions from Author 3, and taking into account all textual suggestions made by the experts. This version was adapted over two rounds until all authors agreed to the next version GRoNC-V1.

*We gratefully acknowledge the following 11 experts (including their source) for their valuable feedback*

1. Wendy de Leng (COTAN)
2. Iris Egberink (COTAN)
3. Jules Ellis (COTAN)
4. Nigel Evans (EFPA Board of assessment)
5. Marjolein Fokkema (COTAN)
6. Bas Hemker (COTAN)
7. John Hunsley (author of chapter)
8. Mark Schittekatte (EFPA Board of assessment)
9. Peter ten Klooster (COTAN)
10. Lieke Voncken (COTAN)
11. Matthias Ziegler (German Diagnostik- Und Testkuratorium)

*Questions asked to the theoretical experts*

1. Are the topics relevant to norming comprehensibly covered? If not, what topic(s) do you miss?

2. Are the topics and questions well-explained and clear? If not, what is insufficiently explained, or unclear?

3. Do you have any other suggestions for the GRoNC?

4. How would you describe your expertise?

### Second round: experts in test development

We asked theoretical experts for feedback on version 1 of the GRoNC. In total, we approached 20 experts, from the network of the authors, including individuals who responded positively upon a call during a talk at the International Test Conference 2024.

In September 2024, we sent out our invitation to participate, including the second version of the GRoNC and four questions, which are provided below. In total 14 experts provided their feedback, who are listed below. The questions were answered by 8 experts, or and three provided a few remarks; extensive suggestions in the text were given by 6 experts.

Author 1 carefully considered all feedback, and incorporated it in the text where deemed appropriate. In case of a substantial comment, she copied the comment next to the text, including reference to the expert number. From these responses, Author 1 extracted 11 points to discuss with Authors 2 and 3. Based on the specific feedback, the team decided to ask for an additional discussion meeting with two test developers, which took place with Author 1. Based the discussions, Authors 1 rewrote GRoNC-V1, with contributions from Author 3, thereby also taking into account all textual suggestions made by the test developers. This version was adapted over three rounds until all authors agreed to on the final GRoNC.

*We gratefully acknowledge the following 14 experts (including a description of their expertise) for their valuable feedback*

1. André Beauducel (test development)
2. Somer Bishop (diagnostic assessment of autism and other neurodevelopmental disorders)
3. Monika Daseking (test development and test adaptation)
4. Reinout de Vries (test developer, teacher on testing)
5. Jan-Phillipp Freudenstein (responsible for the psychometric quality in international assessments at a publisher)
6. Ashley Harrison (lead the development and psychometric evaluations of a measure of autism knowledge and stigma, experienced with standardized testing in clinical practice; teaching about testing)
7. Marcus Hasselhorn (test development)
8. Alexandra Lenhard (test development, test validation, norming)
9. Wolfgang Lenhard (test development, test validation, norming)
10. Simon Nak (responsible for the development, adaptation and/or publication of 40+ tests and questionnaires at a publisher, mostly for clinical purposes)
11. Selma Ruiter (tests development, test adaptation, test standardization, test validation; experienced with standardized testing in clinical practice; teacher on testing)
12. Tanja Sappok (test development and test adaptation)
13. Nicola Taylor (test development, test adaptation, test standardization, co-developer of criteria for test review)
14. Whisnu Yudiana (test publisher, teacher on testing)

*Questions asked to the experts in test development*

1. Are the topics and questions well-explained and clear? If not, what is insufficiently explained, or unclear?

2. Do you think you would be able to write the parts related to norming for a test manual using the GRoNC? If not, could you described what is hampering?

3. Do you have any other suggestions for the GRoNC?

4. How would you describe your expertise?

1. All websites accessed at January 16, 2025. [↑](#footnote-ref-1)
